# Mechanisims of asthma and allergic disease – 1077. Differential expression and roles oF MMP-2, MMP-9, MMP-13, TIMP-1, and TIMP-2 in allergic rhinitis

**DOI:** 10.1186/1939-4551-6-S1-P74

**Published:** 2013-04-23

**Authors:** Sachiko Mori, Ruby Pawankar

**Affiliations:** 1 Otorhinolaryngology, Nippon Medical School, Tokyo, Japan; 2 Allergy and Rhinology, Nippon Medical School, Tokyo, Japan

## Background

Allergic rhinitis (AR) and asthma share many characteristics, but structural changes are observed far less often in AR as compared to asthma and also that in nasal polyps. Matrix metalloproteinases (MMPs) can decompose the extracellular matrix and regulate cell infiltration. In order to understand the role of MMPs in AR, we analyzed the expression of MMPs and their inhibitors, tissue inhibitors of metalloproteinases (TIMPs), in allergic nasal mucosa after nasal allergen challenge (NAC) and determined their relationship to inflammatory cells.

## Methods

Nasal mucosa specimens were obtained at surgery from allergic rhinitis patients. We performed NAC with house dust mite (HDM) allergen disks and control disks, and took biopsies at 30 minutes, 6 hours, and 12 hours after NAC. Cells expressing MMP-2,-9,-13 andTIMP-1,-2, as well as eosinophils and mast cells, were analyzed immunohistochemistry. The MMPs and TIMPs in unchallenged allergic nasal mucosa and nasal polyps were quantified using enzyme-linked immunosorbent assays.

## Results

At 30 minutes post-NAC, HDM-exposed nasal mucosa exhibited significantly more MMP-2,-9,-13 andTIMP-1,-2 positivecells, and the numbers of MMP-9+ and TIMP-1+ cells correlated strongly with the number of mast cells. At 6 hours post-NAC, the ratios of MMPs+ to TIMP-1 were higher in HDM-exposed mucosa. At 12 hours post-NAC, the number of MMP-13+ cells tended to be higher in HDM-exposed mucosa and was strongly correlated with the number of eosinophils. Quantitatively, the levels of MMP-2 and MMP-13 were significantly higher than the MMP-9 level, and the TIMP-2 level was significantly higher than the TIMP-1 level in allergic nasal mucosa. In nasal polyps the levels of MMP-9 were high and TIMPs were low.

## Conclusions

We demonstrated increased expression of MMP-2,-9,-13 in allergic nasal mucosa, high MMPs-to-TIMP-1 ratios, and a strong correlation between MMP-9 and mast cells and between MMP-13 and eosinophils. The imbalance between MMPs and TIMPs may contribute to the migration of inflammatory cells such as eosinophils and mast cells to the nasal mucosa of AR patients, suggesting a possible active role of MMPs in allergic rhinitis. Furthermore, the differential expression of MMPs between AR and nasal polyp may suggest the lack of structural changes in AR versus nasal polyps.

